# Antihyperglycemic and Hypolipidemic Activities of Flavonoids Isolated from Smilax Dominguensis Mediated by Peroxisome Proliferator-Activated Receptors

**DOI:** 10.3390/ph17111451

**Published:** 2024-10-30

**Authors:** Erandi Ortiz-Barragán, Samuel Estrada-Soto, Abraham Giacoman-Martínez, Francisco J. Alarcón-Aguilar, Ángeles Fortis-Barrera, Hugo Marquina-Rodríguez, Emmanuel Gaona-Tovar, Roberto Lazzarini-Lechuga, Alfredo Suárez-Alonso, Julio César Almanza-Pérez

**Affiliations:** 1 Posgrado en Biología Experimental, DCBS, Universidad Autónoma Metropolitana-Iztapalapa, Ciudad de México 09310, Mexico; erandi.ortiz.barragan@gmail.com; 2 Facultad de Farmacia, Universidad Autónoma del Estado de Morelos, Cuernavaca 62209, Mexico; enoch@uaem.mx (S.E.-S.);; 3 Departamento de Ciencias de la Salud, DCBS, Universidad Autónoma Metropolitana-Iztapalapa, Av. Ferrocaril San Rafael Atlixco 186, Col. Leyes de Reforma 1a Secc. Iztapalapa, Ciudad de México 09310, Mexico; a.giacoman@xanum.uam.mx (A.G.-M.); aaaf@xanum.uam.mx (F.J.A.-A.); fortis11_10@yahoo.com.mx (Á.F.-B.); ipn_2nv4@hotmail.com (A.S.-A.); 4 Escuela Superior de Medicina, Instituto Politécnico Nacional, Sección de Estudios de Posgrado e Investigación, Ciudad de México 11340, Mexico; 5 Departamento de Biología de la Reproducción, DCBS, Universidad Autónoma Metropolitana-Iztapalapa, Ciudad de México 09310, Mexico; lazzarini@xanum.uam.mx

**Keywords:** diabetes, hypolipidemic agents, flavonoids, PPARα, PPARγ, *Smilax dominguensis*

## Abstract

**Background/objetives**: Mexican people use Smilax dominguensis as a traditional medicine for diabetes control. Some reports have shown an anti-hyperglycemic effect in animal models. In the current research, a chemical bio-guided fractionation in vitro and in silico was performed to identify compounds with anti-hyperglycemic and hypolipidemic effects through PPARγ/α dual agonist activity because they regulate genes involved in energy storage and burning, such as GLUT4 and FATP. **Methods**: The S. dominguensis extract was evaluated in mice through oral glucose tolerance tests. The bioactive extract was fractionated by open-column chromatography, and seven final fractions (F1–F7) were obtained and evaluated. C2C12 myoblasts were treated with the fractions, and the mRNA expression levels of PPARs, GLUT-4, and FATP were quantified. The most active fractions were evaluated on GLUT-4 translocation and lipid storage in C2C12 cells and 3T3-L1 adipocytes, respectively. **Results**: The F3 fraction increased the expressions of PPARγ, GLUT-4, PPARα, and FATP, and it induced GLUT-4 translocation and decreased lipid storage. F3 was then analyzed by NMR, identifying three flavonoids: luteolin, apigenin, and kaempferol. These compounds were analyzed by molecular docking and on PPAR expressions. Luteolin, apigenin, and kaempferol produced a discrete increase in the mRNA expression of PPARs. Luteolin and kaempferol also decreased lipid storage. **Conclusions**: Our findings indicate that the compounds identified in S. dominguensis exhibit dual agonist activity on PPARγ/PPARα and have the potential for the development of new therapeutic agents helpful in diabetes, obesity, or metabolic syndrome.

## 1. Introduction

Diseases with a metabolic component, such as hypertension, diabetes mellitus, obesity, and dyslipidemia, are becoming more frequent worldwide. These diseases and conditions have a multifactorial origin, and various key molecules play a significant role in their pathophysiology [1]. The absence of a common molecular regulator has made it difficult to design ideal therapeutic strategies. Most therapeutic alternatives are focused on a single molecular target or clinical objective. Drugs used for diabetes aim to lower glucose levels, leaving aside dyslipidemia, while anti-obesity drugs focus on regulating plasma lipid concentration, without significantly modifying glucose levels. Polypharmacy is common in patients, and with it, there is an increased possibility of suffering adverse effects [2]. Therefore, the peroxisome proliferator-activated receptors (PPARs) are considered a key molecular target for developing new drugs to treat different diseases. PPARs function as nuclear transcription factors and include three different types: PPARα (NR1C1), PPARβ/δ (NR1C2), and PPARγ (NR1C3) [3].

PPARγ is generally predominantly in adipose tissue, macrophages, the large intestine, and endothelial cells. It has a key role in glucose balance and fat storage through the expression of proteins like glucose transporter type 4 (GLUT-4) and adiponectin (AdipoQ), among others. The activation of PPARγ by agonist drugs results in an anti-hyperglycemic effect [4,5,6]. On the other hand, PPARα is primarily found in cells or tissues energetically high, such as the skeletal muscle, brown adipose tissue, heart, brain, kidneys, and liver. It plays a significant role in regulating fatty acid oxidation and uptake by influencing the expressions of proteins like the fatty acid transport protein 1 (FATP-1) and acyl-CoA dehydrogenase (AcylCoAD). The activation of PPARα by agonists results in a substantial decrease in lipid storage [7]. Therefore, discovery of dual agonist PPARs would provide a global therapeutic approach, regulating both glucose and lipids [8]. Thus, exploring potential natural components and generating scientific evidence on the effects of medicinal plants are highly relevant.

*Smilax dominguensis*, popularly known as Cocolmeca, China root, Bejuco, or Chiquihuite [9], is used in Latin American traditional medicine for weight loss and as an anti-diabetic remedy. Previous scientific reports have highlighted this species’ anti-obesity and anti-inflammatory properties, which suggest a general beneficial effect on metabolic disorders [10,11]. Additionally, *Smilax dominguensis* has other pharmacological properties, such as antimicrobial, analgesic, and immunomodulatory activities [12,13,14], all which could represent an advantage, in contrast with the drawbacks of other drug treatments used in polypharmacy. Some of its phytochemical compounds have been described, suggesting the presence of alkaloids, such as smilaxin; terpenes, like steroidal saponins and volatile oils; and a variety of phenolic compounds, like pyrocatechol tannins, lignin, quinones, and flavonoids [15]. Although there are pharmacokinetic reports of some metabolites present in the genus *Smilax*, there is still no reported evidence on the pharmacokinetics of *S. dominguensis*. The flavonoids that have been identified comprise flavonols (isorhamnetin and rutin); flavanones, such as naringenin and hesperidin; flavones, such as luteolin and isorhoifolin; flavan-3-ols, such as catechin and gallo-catechin; and flavanonols, such as taxifolin [16]. This group of compounds is relevant because it is thought that they could be potential PPAR agonists [17]. Therefore, *Smilax dominguensis* could possess molecules with potential dual agonist activity to PPARα/γ and could restore homeostasis in metabolic disorders such as diabetes, dyslipidemia, and metabolic syndrome. The objective of this research was to carry out a bio-guided chemical fractionation process aimed at identifying compounds with anti-hyperglycemic properties and dual agonist activity PPARγ/PPARα 

## 2. Results and Discussion

### 2.1. In Vivo Pharmacological Study: Oral Glucose Tolerance Test (OGTT)

Diabetes has a complex physio-pathological mechanism that results in oxidative stress, dyslipidemia, and microvascular and macrovascular complications [18]. Persistent hyperglycemia is the primary factor leading to complications in diabetes, making it a key focus for preventing or mitigating these complications by reducing the associated risks to vascular and other related problems [19].

**Figure 1:**
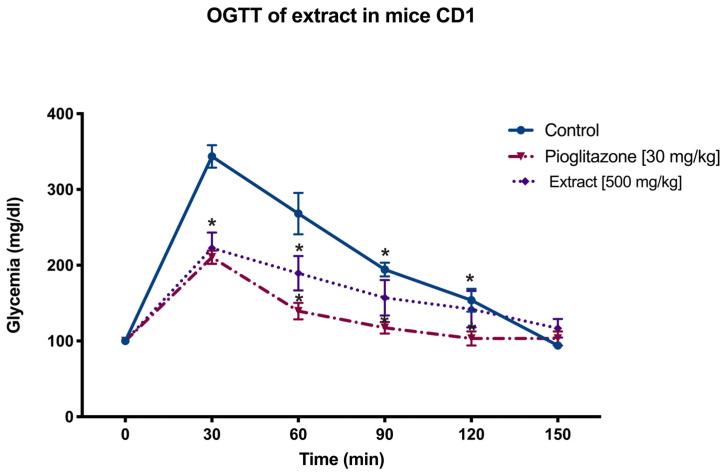
Antihyperglycemic effect of Smilax dominguensis extract CHCl3 on CD1 mice. Oral glucose tolerance tests (OGTTs) were performed in normal male CD1 mice aged 12 weeks. Treatments were administered 20 min before glucose administration, with pioglitazone (5 mg/kg) as the positive control. Mean ± SEM (n = 5). * Significant difference vs. the control group (*p* ≤ 0.05).

An ideal anti-diabetic drug should have the ability to regulate blood glucose through several mechanisms. Nevertheless, drugs with extra-pancreatic effects may offer a long-term advantage by preventing pancreatic exhaustion, a common limitation of secretagogue medications. Bioactive compounds isolated from natural sources are valuable for identifying innovative glucose-lowering drugs [20]. *Smilax* sp. has been used as a traditional anti-diabetic “physick” remedy for many years [21]. Our results showed that, in oral glucose tolerance tests (OGTT), the *S. dominguensis* chloroform extract exerted an anti-hyperglycemic effect (Figure 1). In fact, it inhibited the hyperglycemic peak at 30 and 60 min, compared to the control group. The blood glucose levels decreased at 90 min and normalized at 120–150 min, like pioglitazone, a reference drug that is a PPARγ agonist with an extra-pancreatic mechanism. This effect may be related to insulin sensitization generated by the PPAR activation. Particularly in *S. dominguensis,* some previous reports showed that different flavonoids, like quercetin, rutin, naringenin, and biochanin A, might be good ligands of these receptors [22].

Then, a chemical fractionation guided by bioassay was performed using chromatographic open column to isolate and identify the anti-hyperglycemic compounds. From seven fractions (F1–F7), we found precipitates composed of a mixture of compounds. These fractions were tested in in vitro experiments.

### 2.2. In Vitro Pharmacologic Assays: Effect of Fractions and Compounds on Cell Functionality

The cellular functionality test was performed using the MTT assay. Exponential concentrations (1, 10, and 100 µg/mL) of the extract, fractions (1, 10, and 100 µg/mL), and compounds (1, 10, and 100 µM) were evaluated to determine their non-toxic optimal concentration. The fractions F2, F3, F4, and F5 reduced cellular functionality in less than 85% at 100 µg/mL (Supplementary Data). Therefore, in the following experiments, the concentrations of 10 µg/mL (fractions) and of 10 µM (compounds) were chosen.

#### 2.2.1. In Vitro Pharmacologic Assays: mRNA Expression 

**Figure 2:**
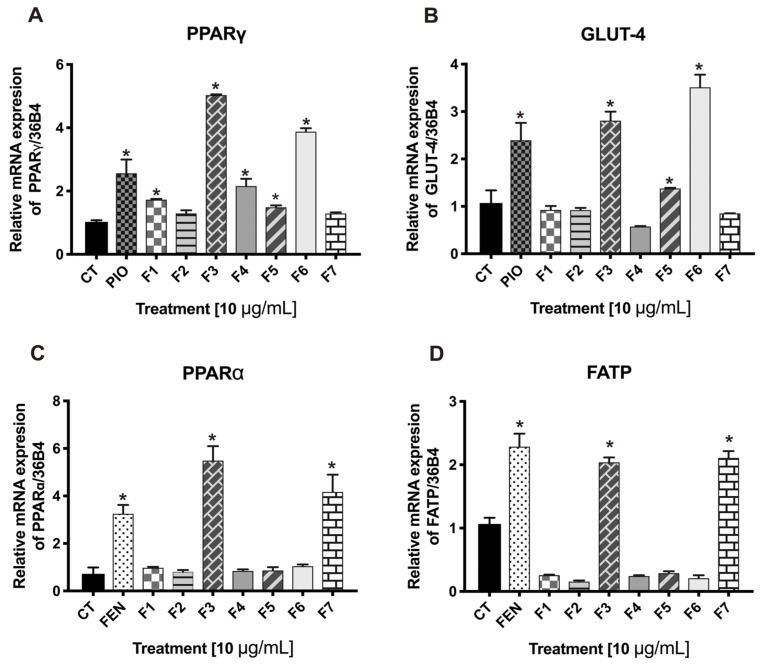
Effects of the fractions on the relative mRNA expression levels of PPARγ (**A**), GLUT4 (**B**), PPARα (**C**), and FATP (**D**) in C2C12 myoblasts. CT = control group. Pioglitazone = synthetic agonist for PPARγ; fenofibrate = agonist for PPARα. Mean ± SEM (n = 6). * Significant difference vs. control (*p* ≤ 0.05).

Based on the findings from the in vivo assessments, we explored the changes induced by the fractions F1 to F7 in the mRNA expressions of PPARγ, PPARα, GLUT4, and FATP. F3 and F6 treatments increased the mRNA expression levels of PPARγ and GLUT-4 against the control group (Figure 2A,B). Interestingly, these fractions generated a greater response than pioglitazone. F5 only increased the mRNA expression levels of PPARγ and GLUT-4 against the control group (Figure 2A,B), below pioglitazone levels. F1 and F4 significantly affected PPARγ; however, they did not affect GLUT-4. In addition, F3 and F7 induced significant gains in PPARα and FATP mRNA expression levels, with respect to the control group, exerting a greater effect on PPARα than fenofibrate (Figure 2C,D). 

These results strongly indicate that *S. dominguensis* may function as a dual agonist of PPARs, which might explain the effects observed in the in vivo tests. A dual agonism associated with PPARs is significant in diabetes control because it reduces the risk of complications, avoids hypoglycemia, and avoids pancreatic exhaustion [23]. On the other hand, PPARs are produced in several tissues, such as the muscle and fatty tissues, known as the tissues with the greatest expression levels. PPARγ regulates the expression of GLUT-4, whereas PPARα regulates FATP [24]. Muscle is significant in metabolism because it is one of the most glucose-demanding tissues; in type 2 diabetes, the muscles develop insulin resistance [25]. Therefore, based on their dual agonist effects on PPARγ and PPARα, fractions F3, F6, and F7 were chosen to continue with their pharmacological characterizations.

#### 2.2.2. In Vitro Pharmacologic Assays: F3, F6, and F7 Inhibit Lipid Storage in 3T3-L1 Adipocytes

Under pathological conditions, the energy balance is affected, leading to triglycerides stored in adipocytes, which causes adipose tissue growth. Hypertrophy and hyperplasia result in overweightness and obesity [26], two risk factors for the establishment of other critical diseases, including diabetes. 

**Figure 3:**
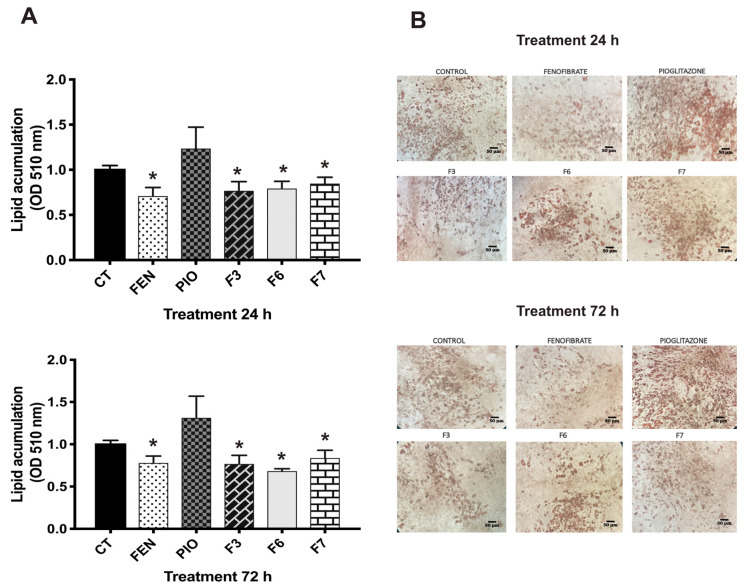
Effect on lipid storage in 3T3-L1 adipocytes. (**A**) Lipid storage was measured after treating the cells with fractions (10 µg/mL), pioglitazone (PIO 5 µM), and fenofibrate (FEN 5 µM) at 24 and 72 h. Lipid storage was detected by optical density (OD) at 510 mm. (**B**) Microphotographs (40×) of lipid droplets on adipocytes using staining with Oil Red following treatment at 24 and 72 h. PIO and FEN were utilized as controls. Mean + SEM (n = 6). * Significant difference vs. control (*p* < 0.05).

F3, F6, and F7 caused significant changes in lipid storage, compared to the control, at 24 h (Figure 3A), which was maintained at 72 h (Figure 3B). Similar activity was observed with the fenofibrate treatment, a PPARα agonist used as a hypolipidemic agent. Fenofibrate reduces triglycerides and increases high-density lipoprotein levels [27]. Furthermore, Oil Red O-stained images corroborated that 3T3-L1 adipocytes treated with these fractions exhibited a reduced intracellular lipid content, which could be associated with the activity observed in PPARα.

#### 2.2.3. In Vitro Pharmacologic Assays: F3 and F6 Treatments Promote GLUT-4 Translocation

**Figure 4:**
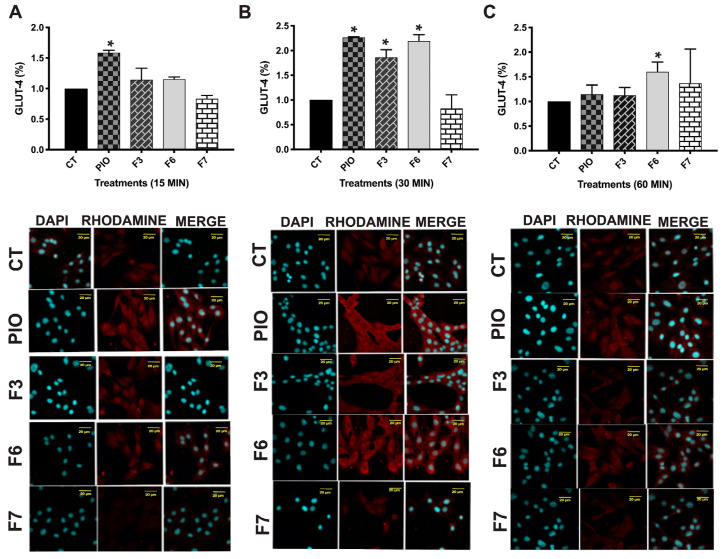
Immunodetection of GLUT-4 translocation in C2C12 myoblasts. Confocal microscopy images illustrate the effects of active fractions (F3, F6, and F7) on GLUT-4 at (**A**) 15 min, (**B**) 30 min, and (**C**) 60 min following treatments (20× magnification). DAPI = nuclear marker. Merged images show the overlap between DAPI and GLUT-4. Additionally, the graphical representation demonstrates the density of the GLUT-4–rhodamine-marked area. Mean + SEM. * Significant difference vs. CT (*p* < 0.05) (n = 3).

PPARγ activation changes the molecular dynamics of GLUT-4, leading to increased mRNA expression and enhanced translocation, ultimately facilitating improved glucose uptake. This process is crucial for glucose homeostasis, which is linked to insulin sensitivity, and it is part of the effects of PPARγ agonists [28]. In this research, immunodetection was detected at 15, 30, and 60 min after the treatment in C2C12 myoblasts treated with F3, F6, and F7 (10 µg/mL) (Figure 4). At 15 min, only pioglitazone exhibited an effect on GLUT-4 translocation (*p* < 0.05). At 30 min, F3 and F6 produced a significant increase. Finally, at 60 min, only F6 maintained an important increase in GLUT-4 translocation, which was slightly higher than the increase observed with pioglitazone. The present research shows that PPARγ activation can enhance GLUT-4 expression and translocation. These findings suggest that F3 may mimic the effects of PPARγ agonists, which can lead to improved metabolic outcomes. Chemical identification was performed on the fraction exhibiting activity throughout the experiments, characterized by the upregulation of PPAR expression, decrease in lipid accumulation, and translocation of GLUT-4.

### 2.3. Phytochemical Characterization

From F3, a yellowish powder was obtained, and luteolin, apigenin, and kaempferol were obtained after several purification processes. These were identified by comparing their H^1^ and ^13^C NMR data with those previously described [29,30,31]. 

### 2.4. In Silico Assays

Some research studies showed that flavonoids have antioxidant, anti-inflammatory, anti-hyperglycemic, anti-hypertensive, and antihyperlipidemic properties [32,33]. 

However, few studies demonstrated the effects of these compounds on lipid storage and PPAR dual agonist activity.

**Figure 5:**
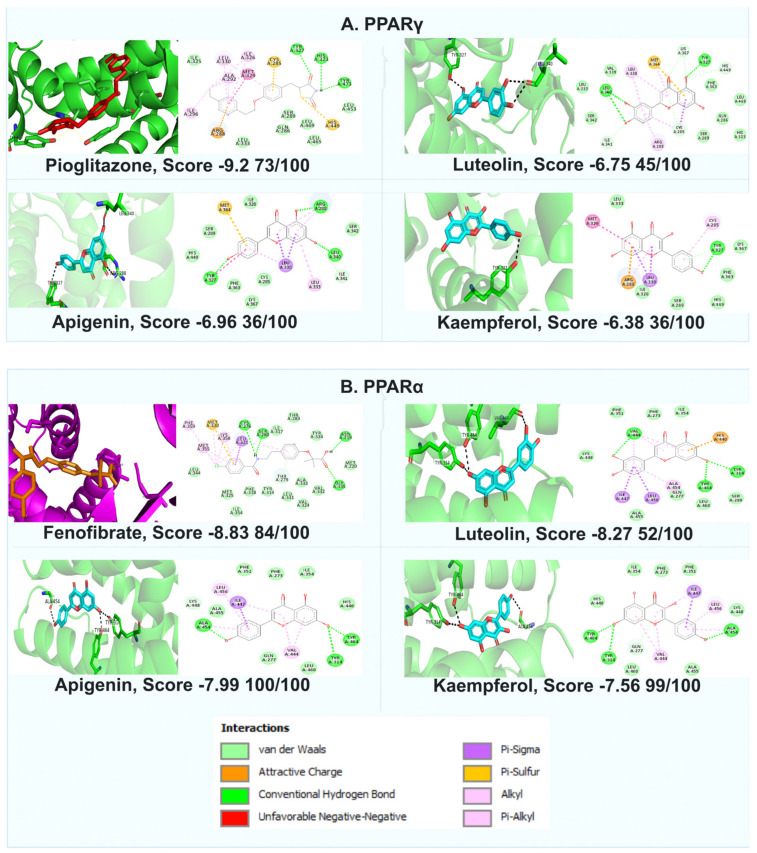
Molecular docking between compounds and PPARs. In silico docked complexes in 3D and 2D. (**A**) Interaction of luteolin, apigenin, and kaempferol in the active site of PPARγ. Pioglitazone = PPARγ agonist, (**B**) Interaction of luteolin, apigenin, and kaempferol with the active site of PPARα and fenofibrate (PPARα agonist). The score is shown in ΔG Kcal/mol, and the same positions per 100 runs are given.

Some previous reports demonstrated that different flavonoids would be suitable ligands for these receptors [34]. Thus, luteolin, apigenin, and kaempferol, flavonoids identified in the active fraction F3, could be responsible for the agonist activity on PPARγ and PPARα. Molecular docking interactions occurred between luteolin–PPARγ by H-bonds with Leu340 and Tyr327; by van der Waals interactions with Phe363, Gln286, and Ser289; and by an attractive charge with Met364, which showed that ΔG = −6.75 Kcal/mol (Figure 5A). On the other hand, results showed that luteolin interacted with PPARα through an H-bond with Val444, Tyr314, and Tyr464; through π-σ interactions with Ile447 and Leu456; and through van der Waals with Phe273, Ile354, and Lys448 (Figure 5B), which showed that ΔG = −8.27 Kcal/mol.

Apigenin–PPARγ showed an interaction through H-bonds with Tyr327, Arg288, and Leu340; π-sulfur with Met364; π-Alkyl with Leu333; and π-σ with Leu330, with ΔG = −6.95 Kcal/mol (Figure 5A). Apigenin–PPARα interacted through H-bonds with Ala454, Tyr464, and Tyr314; π-σ with Ile447; π-alkyl with Val444; and van der Waals with Phe335, Phe273, Ile354, and His440, with ΔG =−7.91 Kcal/mol (Figure 5B).

Kaempferol interacted with PPARγ through H-bonds in the residues with Tyr327; π-alkyl with Leu330 and Cys285; π-σ with Met329; and van der Waals with Ile326, Ser289, His449, Phe363, and Lys367, with ΔG = −6.38 Kcal/mol (Figure 5A). The kaempferol–PPARα interaction was through H-bonds with Tyr464, Tyr314, and Ala454; π–σ with Ile447; π-alkyl with Val444; and van der Waals with residues of His440, Leu460, Ile354, Phe273, and Phe351, with ΔG = −7.56 Kcal/mol (Figure 5B). The three flavonoids showed identical interactions through H-bonds with Tyr327 in PPARγ in the same way as pioglitazone, which could be a key interaction in activating this nuclear receptor. Fenofibrate, a PPARα agonist, showed a strong interaction through H-bonds with Cys276, Ser280, Asn219, and Gly335. On the other hand, the three compounds evaluated did not show the same H-bonds. However, they interacted with Tyr314 in all cases, which could be an important amino acid in activating this receptor; however, this hypothesis must be investigated in further studies.

### 2.5. In Vitro Pharmacologic Assays: Luteolin and Kaempferol Promote Increases in the mRNA Expression Levels of PPARγ, FATP, and GLUT-4

**Figure 6:**
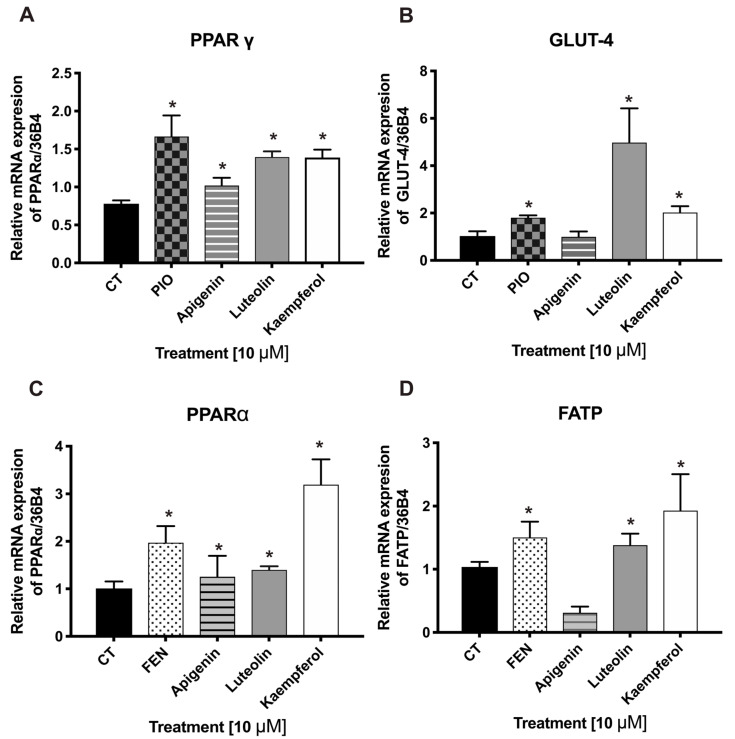
Effects of apigenin, luteolin, and kaempferol on the mRNA expressions of PPARγ (**A**), GLUT-4 (**B**), PPARα (**C**), and FATP (**D**) in C2C12 myoblasts. Pioglitazone (PIO) is a drug agonist of PPARγ, and fenofibrate (FEN) is a drug agonist of PPARα. Mean ± SEM (n = 6). * Significant difference vs. the control (*p* ≤ 0.05).

To validate the findings from the in silico analysis, we evaluated luteolin, apigenin, and kaempferol in C2C12 myoblasts to determine the effects on the mRNA expressions of PPARs, GLUT-4, and FATP. The treatments with luteolin, kaempferol, and apigenin increased the mRNA expression levels of PPARγ and PPARα against the control group (Figure 6A–C), while the gene expression levels of GLUT-4 and FATP were increased only with luteolin and kaempferol, compared to the control group (Figure 6B–D).

#### In Vitro Pharmacologic Assays: Luteolin and Kaempferol Inhibit Lipid Storage in 3T3-L1 Adipocytes

**Figure 7:**
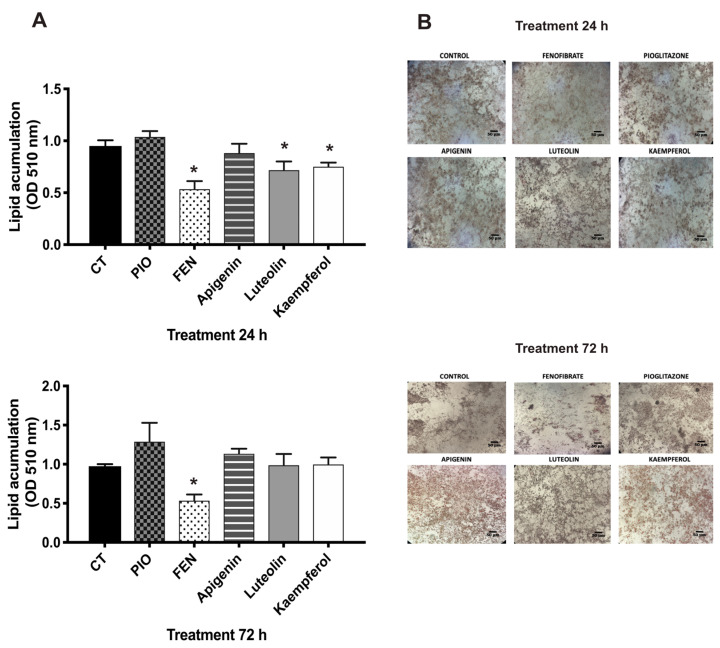
Effects of compounds on lipid storage in 3T3-L1 adipocytes. (**A**) Lipid storage was measured after treating the cells with apigenin, luteolin, and kaempferol (10 µM); pioglitazone (PIO 5 µM), and fenofibrate (FEN 5 µM) at 24 and 72 h. Lipid storage was detected by optical density (OD) at 510 mm. The data were processed using the control group data. (**B**) Microphotographs (40×) of lipid droplets on adipocytes using Oil Red staining following treatment at 24 and 72 h. PIO and FEN were utilized as positive controls. Mean + SEM (n = 6). * Significant difference vs. control (*p* ≤ 0.05).

The lipid-lowering effect attributed to the presence of flavonoids could be due to different mechanisms; it has been shown that flavonoids protect against the oxidation of low-density lipoproteins. It is also known that some flavonoids regulate the lipids in diabetes and hypercholesterolemia through PPAR activity agonists [35,36]. We evaluated the effects of the three flavonoids, observing that kaempferol and luteolin reduced lipid storage at 24 h, compared to the control, but at 72 h, lipid storage remained unchanged (Figure 7A,B). There were no significant differences, compared to the control group, but we detected a slight decrease, compared to pioglitazone. The in silico studies revealed that they might act as dual agonists of PPARγ/α with a good affinity energy and could be related to and involved with the effects on lipid storage and GLUT-4 translocation. Notably, the effect generated by fraction F3 was greater than that recorded by the individually evaluated compounds, which may reflect a possible synergy among the compounds. Future studies could be directed toward evaluating the compound mixture. These data suggest that these compounds possess properties for new multitarget interactions, which should be key for designing novel potential compounds.

## 3. Materials and Methods

### 3.1. Plant Material and Extract Preparation

*S. dominguensis* was collected in San Agustín Loxicha, Oaxaca, Mexico. The vegetal material was identified by comparison with the specimen catalogued in The National Herbarium of Mexico (voucher Nos. 586067 and 1198144). The root of *S. dominguensis* was dried and ground at room temperature. We used grinder (2 mm mesh) of model 4 (Wiley, Swedesboro, NJ, USA). Then, it was subjected to soxhlet extraction with chloroform 99.9% (Merck, Rahway, NJ, USA) (500 mL/50 g of the dry plant), and the extract was filtered. The extract was subjected to evaporation in a rotary evaporator (Buchi Labortechnik, Flawil, Switzerland) to concentrate, and finally, the traces of solvent were evaporated in a laminar flow hood.

### 3.2. Fractionation of the Extract of S. dominguensis

The chloroform extract (6 g) was subjected to fractionation using open column chromatography (900 × 50 mm) with silica gel 60 (0.2–0.5 mm, 250 g; Merck, USA). A gradient system employing n-hexane and dichloromethane (Fermont, Monterrey, NL, México) was utilized as the mobile phase; the process started with 100% n-hexane and dichloromethane was progressively added. Once the concentration of dichloromethane reached 100%, the gradient was continued with ethyl acetate, followed by methanol in a similar manner. Each sample volume was 100 mL, and there were partial increments of 10% solvent. This process was performed for each sample, and 5 fractions were taken. Once 100% was reached, the procedure was completed. From the column, 515 fractions were obtained, and each fraction was analyzed by thin-layer chromatography (TLC) in normal phase (F_254_ 20 × 20 cm of aluminum sheets with silica gel prepared with fluorescent indicator). Different detection methods were used (wavelengths short at 254 nm; wavelengths long at 365 nm, with ceric sulfate). Then, the fractions were rearranged by chemical composition similarity, resulting in 21 final fractions by TLC. From these, 7 fractions precipitated spontaneously (designated from F1 to F7). These fractions were subjected to further purification, characterized by nuclear magnetic resonance (NMR) spectroscopy, and evaluated in subsequent experiments.

### 3.3. In Vivo Assay

#### Animals

Male CD-1 mice weighing between 30–35 g at 6–8 weeks of age were used. These animals were obtained from the UMADI (Animal Handling Unit for Teaching and Research) at the Metropolitan Autonomous University. The animals were given a standard rodent diet (Harlan Laboratories, Indianapolis, IN, USA) and had access to water ad libitum, maintained under a 12 h light/12 h dark cycle. The experimental protocol adhered to the International Guidelines for the Care and Use of Laboratory Animals (NOM-062-ZOO-1999, 2001 revision). An oral glucose tolerance test (OGTT) was made to evaluate the anti-hyperglycemic effect. The experimental treatments were administered 20 min before oral glucose administration (2 g/kg): control Tween 20 at 5% (Karal S. A. de CV, León, Mexico), pioglitazone (20 mg/kg), and the experimental group (extract 500 mg/kg). Glycemia was measured with an Accu-Chek Performa glucometer (Roche, Mexico City, México) at 0, 30, 60, 90, 120, and 150 min.

### 3.4. In Vitro Pharmacologic Assays

#### 3.4.1. C2C12 Myoblasts Culture

ATCC CRL-1772 cells were grown in 75 cm^3^ culture flasks (Life Science, Gyeonggi-do, Republic of Korea) using DMEM (ATCC 30-2002), and cultures were maintained as described by Loza-Rodriguez et al., 2020 [9]. 

#### 3.4.2. 3T3-LI Fibroblasts Culture

ATCC CL-173 cells were grown in 75 cm^3^ flasks (Life Science, Gyeonggi-do, Republic of Korea) using DMEM as described by Cave and Crowther, 2018 [37]. That medium was refreshed every two days. When the cells reached confluence (day 0), differentiation was used. This medium contained 0.5 mM methyl-isobutyl-xanthine (MIX), dexamethasone 0.25 µM (DX), and human insulin 0.8 µM in DMEM, with 10% FBS [38,39]. Culture and differentiation conditions were carried out as described by Contreras-Nuñez et al., 2018 [40].

#### 3.4.3. Cell Functionality

The MTT assay (3-(4,5-dimethylthiazole-2-yl)-2,5-diphenyltetrazolium bromide) (NR, Sigma-Aldrich, St. Louis, MO, USA) was employed to evaluate cellular functionality as described by Mosmann et al., 1983 [41]. Cells were cultivated in 96-well microplates as described by Loza et al., 2020 [9]. Several concentrations of fractions F1 to F7 (1, 10, and 100 µg/mL) and compounds (1, 10, and 100 µM) were applied for 24 h (n = 4). At 570 nm, the absorbance was measured.

#### 3.4.4. mRNA Expression Analysis of PPARγ, GLUT-4, PPARα, and FATP 

The cells were treated with fractions F1 to F7 (10 µg/mL) or compounds (10 µM) for 24 h, pioglitazone (reference drug for PPARγ; 5 µM), and fenofibrate (reference drug for PPARα; 5 µM) dissolved in DMEM. Following the treatments, RNA was isolated by the TRizol method (Invitrogen, Los Angeles, CA, USA) [42].

Total RNA was reverse transcribed (RT) using the ImProm II kit (Promega, Madison, WI, USA) [18]. One microgram of RNA was run using the electrophoresis method into a 2% agarose gel dyed with Eco-Stain colorant (BIOBASIC, Markham, ON, Canada). This gel was visualized on an imaging analyzer (BioRad, Redwood City, CA, USA) to confirm the RNA integrity. The complementary DNA (cDNA) was used to perform the PCR using SYBR Green (Thermo Scientific, Waltham, MA, USA) with specific primers (Table 1). The expression level quantification was made as described by Giacoman-Martínez et al., 2017 [4].

#### 3.4.5. GLUT-4 Translocation

The C2C12 myoblasts were used as described by Loza-Rodríguez et al., 2020 [9]. The cells were treated with 10 µg/mL active fractions, which significantly increased the expression levels of PPARs and their regulated genes: F3, F5, and F6 for 15, 30, and 60 min. For the immunodetection of GLUT-4 translocation, we used a primary antibody GLUT-4 (Santa Cruz Biotechnology, Dallas, TX, USA) and a secondary antibody anti-rabbit-rhodamine (Santa Cruz Biotechnology, TX, USA). For detection, we used the methodology described by Loza-Rodríguez et al., 2020 [9]. We used a Zen-Sp1 ZEISS confocal microscope with laser scanning for images captured (Oberkochen, Germany). Several fields were selected, and we obtained the average pixel intensity using ImageJ software version 1.53 (Bethesda, MD, USA).

#### 3.4.6. Oil Red Staining

The 3T3-L1 fibroblasts were treated with fractions F1 to F7 (10 µg/mL) or compounds (10 µM) for 24 h, pioglitazone (reference drug for PPARγ; 5 µM), and fenofibrate (reference drug for PPARα; 5 µM). After the treatments, the cells were washed and fixed for 30 min. A solution of 1.2 mg/mL of Oil Red O dye in 60% isopropanol (Sigma-Aldrich, Darmstadt, Germany) was used for cells stained for 10 min. Following the staining, the O-red dye was secluded using ethanol and PBS. Isopropanol containing 4% Nonidet P-40 was used to extract the O-red dye from the adipocytes and quantified using spectrometry at 510 mm. Finally, lipid storage was quantified using normalized control data [43,44].

### 3.5. In Silico Assays

Molecular docking was made with the AutoDock software. PPARγ and PPARα crystal structures were obtained from the PDB (code 1I7G and 1I7I, respectively). The predictions were carried out using AutoDock version 4.2.6, following the procedures outlined by Hidalgo-Figueroa et al., 2017 [45]. A grid of dimensions of 50 × 50 × 50 points was created, with a spacing of 0.375 Å points. The Lamarckian genetic algorithm with default parameters was employed for the search, and 100 docking runs were performed. After docking, the results were clustered based on ΔG = Kcal/mol. The results in 2D and 3D were visualized using Discovery Studio version 3.5 (Accelrys, Inc., San Diego, CA, USA) and PyMOL version 2.7 (Schrodinger software). Validation of docking was executed in AutoDock 4.2.6 using PDB proteins 1I7G (PPARα) and 1I7I (PPARγ). The structure was constructed and analyzed in the active site using the X-ray crystal, and validation was performed using co-crystal ligands and proteins to ensure the reproducibility of molecular docking studies with both receptors.

## 4. Conclusions

The bio-guided chemical fractionation of *Smilax dominguensis* allowed the obtention of dual PPAR agonist fractions and the identification of pharmacologically active compounds (luteolin, apigenin, and kaempferol) from the F3 fraction. The anti-hyperglycemic effect and reduction of lipid storage by *Smilax dominguensis* can be associated with PPARγ/agonist activity and GLUT-4 translocation. The identified flavonoids involved with these pharmacological effects could be important in energy balance. The present study establishes the basis for developing a standardized phytomedicine with anti-hyperglycemic and hypolipidemic activities, with the capacity to reduce the polypharmacy used in the treatment of metabolic diseases.

## Figures and Tables

**Table 1:** Specific oligonucleotides used in qPCR for *PPARγ*, *GLUT-4*, *PPARα*, *FATP*, 36B4.

Gene	Primer	Gene Bank
**PPARγ**	**Forward** 5′-CCAGAGTCTGCTGATCTGCG-3′	NM_011146.1
	**Reverse** 5′-GCCACCTCTTTGCTCTGCTC-3′	
**GLUT-4**	**Forward** 5′-GATTCTGCTGCCCTTCTGTC-3′	NM_009204.2
	**Reverse** 5′-ATTGGACGCTCTCTCTCCAA-3′	
**PPARα**	**Forward** 5′-TGGAGCTCGATGACAGTGAC-3′	NM011145
	**Reverse** 5′-GTACTGGCTGTCAGGGTGGT-3′	
**FATP-1**	**Forward** 5′-ACCAGTGTCCAGGGGTACAG-3′	NM011977.3
	**Reverse** 5′-TGTCTCCCAGCTGACATGAG-3′	
**36B4**	**Forward** 5′-AAGCGCGTCCTGGCATTGTCT-3′	NM_007475.2
	**Reverse** 5′-CCGCAGGGGCAGCAGTGGT-3′	

## Data Availability

The original contributions presented in this study are included in the article; further inquiries can be directed to the corresponding authors.

## Supplementary Materials

The supplementary information can be downloaded at: https://www.mdpi.com/article/10.3390/ph17111451/s1. Supplementary S1. Effect of precipitate fractions on cell functionality. Supplementary S2. Effect of mixture compounds on PPARγ, PPARα, GLUT-4, and FATP mRNA expression. Supplementary S3. 1H Nuclear Magnetic Resonance Spectral Data (ppm) in Purified precipitate of Fraction F3.
