# Supplementary material for: Antihyperglycemic and Hypolipidemic Activities of Flavonoids Isolated from Smilax Dominguensis Mediated by Peroxisome Proliferator-Activated Receptors
Source: Pharmaceuticals (Basel). 2024 Oct 30;17(11):1451. doi: 10.3390/ph17111451 (PMC11597028; doi:10.3390/ph17111451)
Supplement: Supplementary file 1 [file pharmaceuticals-17-01451-s001.zip › pharmaceuticals-3265401-supplementary.pdf]

pharmaceuticals

Supplementary S1. Effect of precipitate fractions on cell functionality.

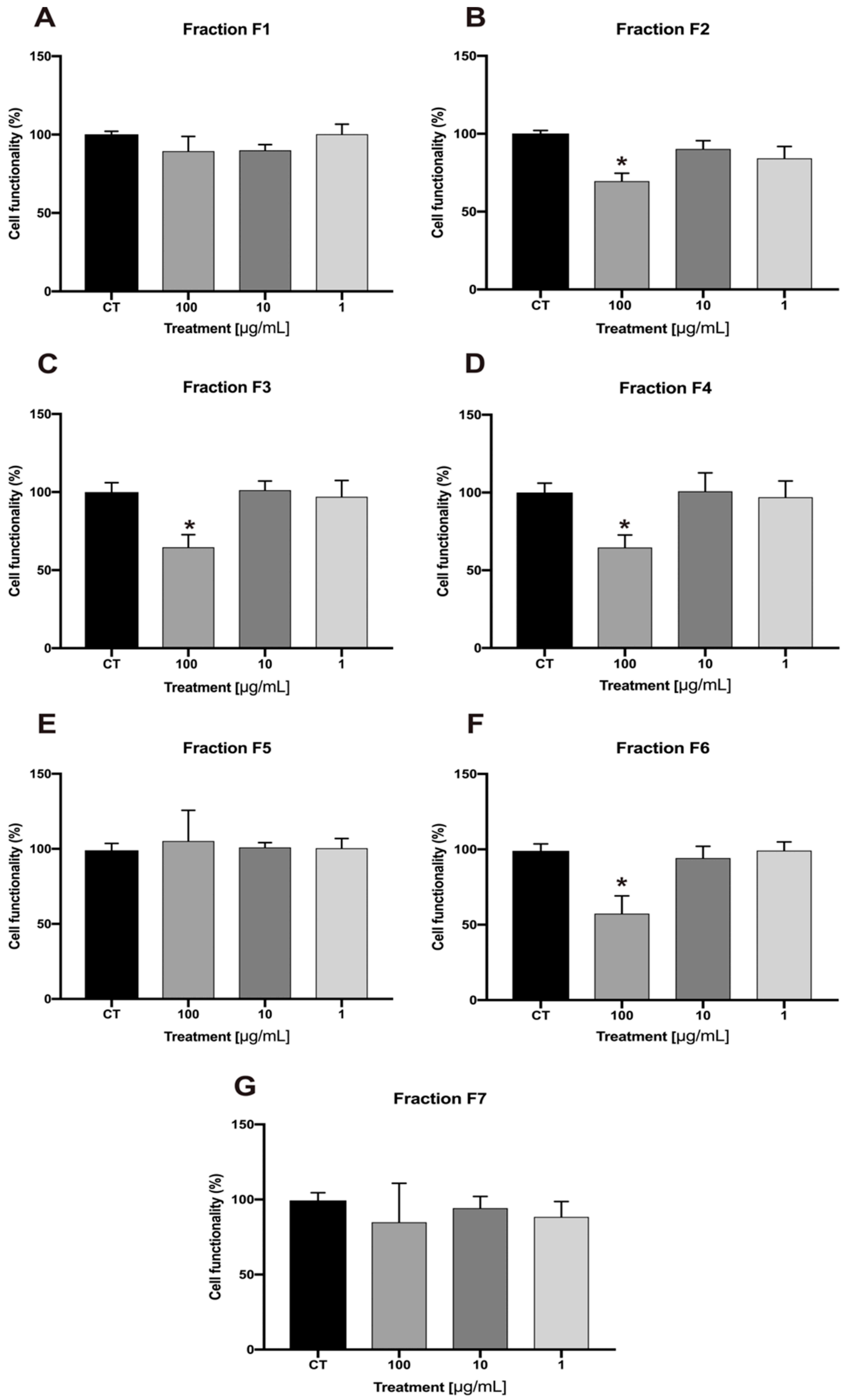

Figure S1. Effect of precipitated fractions on cell functionality of C2C12 myoblasts. Cells were treated with 1, 10, or 100 µg/mL by 24 h, and functionality was quantified by MTT assay. Mean ± SEM. (n = 8). CT: control. *Significant difference vs. the control ($p < 0.05$).

Supplementary S2. Effect of mixture compounds on PPARγ, PPARα, GLUT-4, and FATP mRNA expression.

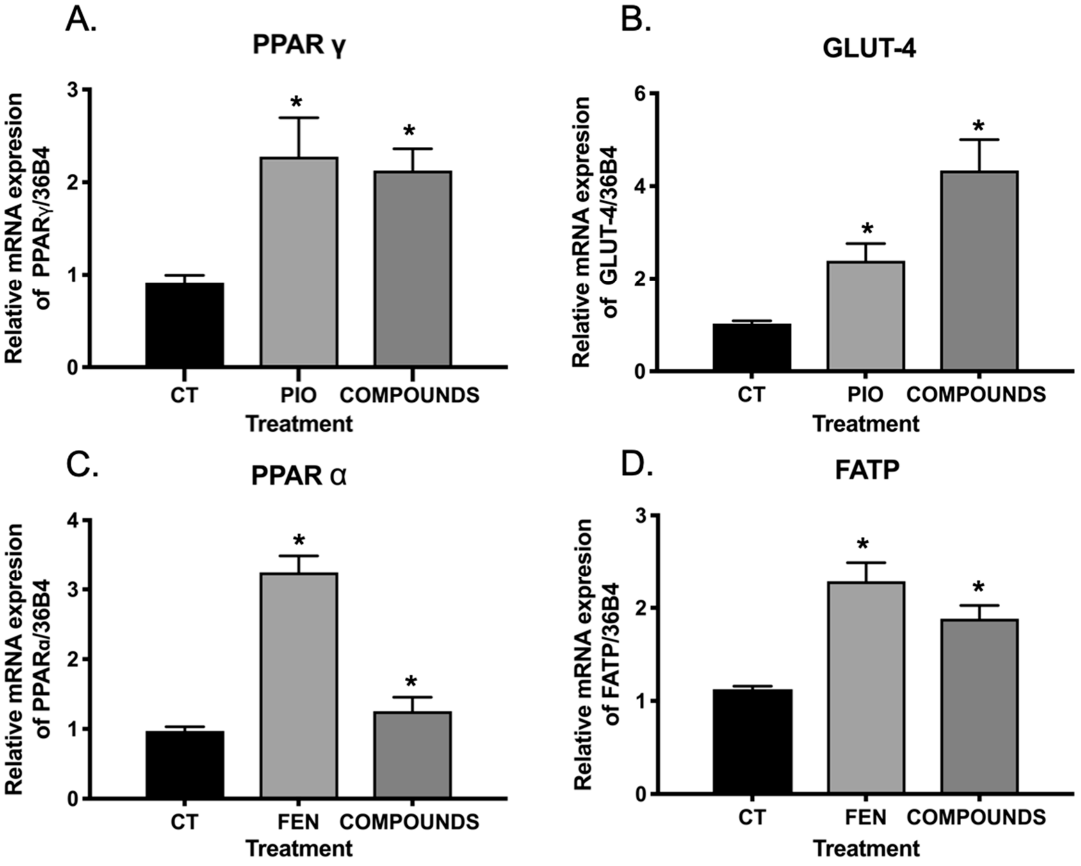

Figure S2. Effect of compounds in an equimolar mixture (apigenin, luteolin, and kaempferol) on the mRNA expression of PPARγ (A), GLUT-4 (B), PPARα (C), and FATP (D) in C2C12 myoblasts. Pioglitazone (PIO) is a synthetic agonist of PPARγ, and Fenofibrate (FEN) is a synthetic agonist of PPARα. Mean ± SEM (n = 6). *Significant difference vs. the control ($p \leq 0.05$).

Supplementary S3. ^{1}H Nuclear Magnetic Resonance Spectral Data (ppm) in Purified precipitate of Fraction F3

Luteolin

^{1}H NMR (400 MHz, CDCl 3): δ 7.00 (d, J = 13.5 Hz, 2H), 6.73 (s, 1H), 4.78 (s, 1H), 4.24 (s, 1H), 3.81 (s, 1H), (s,1H), 6.56 (s, 1H), 6.18 (s, 1H), 6.05 0.40 (s, 1H).

Apigenin

^{1}H NMR (500 MHz, in DMSO-d6): δ 6.75 (s, 1H, H-3), 6.15 (d, 1H, J= 1,95 Hz, H-5), 6.44 (d, 1H, J= 1,95 Hz, H-8), 7.91 (d, 2H, J= 9,05 Hz, H-2′/H-6′), 6.90 (d, 2H, J= 9,05 Hz, H-3′/H-5′), 12.94 (-OH).

Kaempferol

^{1}H NMR (CD3OD, 400 MHz): δ 8.05(2H, d, J=8.5 Hz), 6.88 (2H, d, J=8.5 Hz), 6.35 (1H, s), 6.15(1H, s).
